# Improvement of Bobrovsky–Mayor–Wolf–Zakai Bound

**DOI:** 10.3390/e23020161

**Published:** 2021-01-28

**Authors:** Ken-ichi Koike, Shintaro Hashimoto

**Affiliations:** 1 College of Commerce, Nihon University, Tokyo 157-8570, Japan; 2 Department of Mathematics, Hiroshima University, Hiroshima 739-8521, Japan; s-hashimoto@hiroshima-u.ac.jp

**Keywords:** Bayes risk, Bobrovsky–Mayor–Wolf–Zakai bound, Van Trees bound, Borovkov–Sakhanenko bounds, Laplace approximation

## Abstract

This paper presents a difference-type lower bound for the Bayes risk as a difference-type extension of the Borovkov–Sakhanenko bound. The resulting bound asymptotically improves the Bobrovsky–Mayor–Wolf–Zakai bound which is difference-type extension of the Van Trees bound. Some examples are also given.

## 1. Introduction

The Bayesian Cramér–Rao bound or Van Trees bound [1] has been extended in a number of directions (e.g., [1,2,3]). For example, multivariate cases for such bounds are discussed by [4]. These bounds are used in many practical fields such as signal processing and nonlinear filtering. However, these bounds are not always sharp. To improve them, Bhattacharrya type extensions for them were provided by [5,6]. These Bayesian bounds are split into two categories, the Weiss–Weinstein family [7,8,9] and the Ziv–Zakai family [10,11,12]. The work in [13] serves as an excellent reference of this topic.

Recently, the authors in [14] showed that the Borovkov–Sakhanenko bound is asymptotically better than the Van Trees bound, and asymptotically optimal in a certain class of bounds. The authors in [15] compared some Bayesian bounds from the point of view of asymptotic efficiency. Furthermore, necessary and sufficient conditions for the attainment of Borovkov–Sakhanenko and the Van Trees bounds were given by [16] for an exponential family with conjugate and Jeffreys priors.

On the other hand, the Bobrovsky–Mayor–Wolf–Zakai bound ([17]) is known as a difference-type (Chapman–Robbins type) variation of the Van Trees bound. In this paper, we consider the improvement of the Bobrovsky–Mayor–Wolf–Zakai bound by applying the Chapman–Robbins type extension of the Borovkov–Sakhanenko bound. This bound is categorized into Weiss–Weinstein family.

As discussed later, the obtained bound is asymptotically superior to the Bobrovsky–Mayor–Wolf–Zakai bound for a sufficiently small perturbation and large sample size. We also provide several examples for finite and large sample size settings which include conjugate normal and Bernoulli logit models.

## 2. Improvement of Bobrovsky–Mayor–Wolf–Zakai Bound

Let $X_{1},\ldots ,X_{n}$ be a sequence of independent, identically distributed (iid) random variables with density function $f_{1}\left(x\;|\;\theta \right)$$\left(\theta \in \Theta =R^{1}\right)$ with respect to a σ-finite measure μ. Suppose that $f_{1}\left(x\;|\;\theta \right)$ is twice partial differentiable with respect to θ, and support $\left\{x\;|\;f_{1}\left(x\;|\;\theta \right)>0\right\}$ of $f_{1}\left(x\;|\;\theta \right)$ is independent of θ. The joint probability density function of $X:=(X_{1},\ldots ,X_{n})$ is $f\left(x\;|\;\theta \right):=\prod_{i=1}^{n}f_{1}\left(x_{i}\;|\;\theta \right)$, where $x=(x_{1},\ldots ,x_{n})$. Let λ(θ) be a prior density of θ with respect to the Lebesgue measure. Consider the Bayesian estimation problem for a function φ(θ) of θ under quadratic loss $L\left(\theta ,a\right)={\left(a-\varphi \left(\theta \right)\right)}^{2}$. The joint pdf f(x,θ) of (X,θ) is given by f(x,θ)=f(x|θ)λ(θ). Hereafter, expectations under probability densities f(x,θ) and f(x|θ) are denoted by E(·) and $E_{\theta }\left(\cdot \right)$, respectively. We often use prime notation for partial derivatives with respect to θ for brevity, for example, $\frac{\partial }{\partial \theta }\varphi \left(\theta \right)$ is expressed as $\varphi^{\prime }\left(\theta \right)$.

In this paper, we assume the following regularity conditions (A1)–(A3).

(A1) φ(θ) is twice differentiable.
(A2) Fisher information number
  $$0<I\left(\theta \right)=-E_{\theta }\left\{\partial^{2}\log f_{1}\left(X_{1}|\theta \right)/\partial \theta^{2}\right\}=E_{\theta }\left[{\left\{\partial \log f_{1}\left(X_{1}|\theta \right)/\partial \theta \right\}}^{2}\right]<\infty$$
  for arbitrary θ∈Θ and is continuously differentiable in Θ.
(A3) Prior density λ(θ) of θ is positive and differentiable for arbitrary θ∈Θ and
  $\lim_{\theta \to \pm \infty }\lambda \left(\theta \right)=0$.

Let $G_{h}=\frac{1}{h}\left(\frac{f(x,\theta +h)}{f(x,\theta )}\frac{\varphi^{\prime }\left(\theta +h\right)}{I(\theta +h)}-\frac{\varphi^{\prime }\left(\theta \right)}{I(\theta )}\right)$. Considering variance–covariance inequality for $G_{h}$, we have the following theorem for the Bayes risk.

**Theorem** **1.** *Assume (A1)–(A3). For an estimator $\hat{\varphi }\left(X\right)$ of φ(θ) and a real number h, inequality*

(1) $$E\left\{{\left(\hat{\varphi }\left(X\right)-\varphi \left(\theta \right)\right)}^{2}\right\}\geq \frac{{\left\{\mathrm{Cov}(G_{h},\hat{\varphi }\left(X\right)-\varphi \left(\theta \right))\right\}}^{2}}{E(G_{h}^{2})}=\frac{{\left\{E\left[\left\{\varphi \left(\theta \right)-\varphi \left(\theta -h\right)\right\}\frac{\varphi^{\prime }\left(\theta \right)}{I(\theta )}\right]\right\}}^{2}}{E\left[{\left\{\frac{f(X,\theta +h)}{f(X,\theta )}\frac{\varphi^{\prime }\left(\theta +h\right)}{I(\theta +h)}-\frac{\varphi^{\prime }\left(\theta \right)}{I(\theta )}\right\}}^{2}\right]}$$

*for the Bayes risk holds.*

Bound (1) is directly derived as a special case of the Weiss–Weinstein class [7]. However, we prove it in the Appendix B for the sake of clarity.

Note that

(2) $$\begin{array}{cc} \lim_{h\to 0}G_{h}= & \frac{1}{f(x,\theta )}\lim_{h\to 0}\frac{1}{h}\left\{f\left(x,\theta +h\right)\frac{\varphi^{\prime }\left(\theta +h\right)}{I(\theta +h)}-f\left(x,\theta \right)\frac{\varphi^{\prime }\left(\theta \right)}{I(\theta )}\right\}\\ = & \frac{1}{f(x,\theta )}\frac{\partial }{\partial \theta }\left\{f\left(x,\theta \right)\frac{\varphi^{\prime }\left(\theta \right)}{I(\theta )}\right\}\left(=G_{0},\mathrm{say}\right). \end{array}$$

The Borovkov–Sakhanenko bound is obtained from the variance–covariance inequality for $G_{0}$

(3) $$E\left\{{\left(\hat{\varphi }\left(X\right)-\varphi \left(\theta \right)\right)}^{2}\right\}\geq \frac{{\left\{\mathrm{Cov}(G_{0},\hat{\varphi }\left(X\right)-\varphi \left(\theta \right))\right\}}^{2}}{E(G_{0}^{2})}=\frac{{\left\{E\left(\frac{{\varphi^{\prime }\left(\theta \right)}^{2}}{I\left(\theta \right)}\right)\right\}}^{2}}{nE\left(\frac{{\varphi^{\prime }\left(\theta \right)}^{2}}{I\left(\theta \right)}\right)+E\left[{\left\{\frac{{\left(\varphi^{\prime }\left(\theta \right)\lambda \left(\theta \right)/I\left(\theta \right)\right)}^{\prime }}{\lambda \left(\theta \right)}\right\}}^{2}\right]}$$

([2]). Since Bound (1) converges to Bound (3) as h→0 under Condition (B1) in Appendix A, Bound (1) for a sufficiently small *h* is very close to Bound (3).

In a similar way, the Bobrovsky–Mayor–Wolf–Zakai bound is obtained from variance–covariance inequality

(4) $$E\left\{{\left(\hat{\varphi }\left(X\right)-\varphi \left(\theta \right)\right)}^{2}\right\}\geq \frac{{\left\{\mathrm{Cov}(B_{h},\hat{\varphi }\left(X\right)-\varphi \left(\theta \right))\right\}}^{2}}{E(B_{h}^{2})}=\frac{{\left[E\{\varphi (\theta )-\varphi (\theta -h)\}\right]}^{2}}{E\left[{\left\{\frac{f(X,\theta +h)}{f(X,\theta )}\right\}}^{2}\right]-1},$$

where $B_{h}=\frac{1}{h}\left(\frac{f(x,\theta +h)}{f(x,\theta )}-1\right)$ ([17]). By applying $\lim_{h\to 0}B_{h}=B_{0}=\frac{\frac{\partial }{\partial \theta }f\left(x,\theta \right)}{f(x,\theta )}$ to the variance–covariance inequality, we have the Van Trees bound, that is,

(5) $$E\left\{{\left(\hat{\varphi }\left(X\right)-\varphi \left(\theta \right)\right)}^{2}\right\}\geq \frac{{\left\{\mathrm{Cov}(B_{0},\hat{\varphi }\left(X\right)-\varphi \left(\theta \right))\right\}}^{2}}{E(B_{0}^{2})}=\frac{{\left\{E\left(\varphi^{\prime }\left(\theta \right)\right)\right\}}^{2}}{nE\left\{I(\theta )\right\}+E{\left\{\frac{\lambda^{\prime }\left(\theta \right)}{\lambda (\theta )}\right\}}^{2}}.$$

Since $\lim_{h\to 0}B_{h}=B_{0}$, the value of Bobrovsky–Mayor–Wolf–Zakai Bound (4) converges to Van Trees Bound (5) as h→0 under (B2) in Appendix A. Hence, the value of Bound (4) for a sufficiently small *h* is very close to the one of Bound (5) in this case.

On the other hand, we often consider the *normalized* risk

(6) $$\lim_{n\to \infty }nE\left\{{\left(\hat{\varphi }\left(X\right)-\varphi \left(\theta \right)\right)}^{2}\right\}$$

(see [3,14]). For the evaluation of the normalized risk (6), Bayesian Cramér–Rao bounds can be used. For example, from Bound (3),

(7) $$\lim_{n\to \infty }nE\left\{{\left(\hat{\varphi }\left(X\right)-\varphi \left(\theta \right)\right)}^{2}\right\}\geq \lim_{n\to \infty }n\frac{{\left\{E\left(\frac{{\varphi^{\prime }\left(\theta \right)}^{2}}{I\left(\theta \right)}\right)\right\}}^{2}}{nE\left(\frac{{\varphi^{\prime }\left(\theta \right)}^{2}}{I\left(\theta \right)}\right)+E\left[{\left\{\frac{{\left(\varphi^{\prime }\left(\theta \right)\lambda \left(\theta \right)/I\left(\theta \right)\right)}^{\prime }}{\lambda \left(\theta \right)}\right\}}^{2}\right]}=E\left(\frac{\varphi^{\prime }{\left(\theta \right)}^{2}}{I(\theta )}\right).$$

Moreover, the authors in [14,15] showed that the Borovkov–Sakhanenko bound is asymptotically optimal in some class, and asymptotically superior to the Van Trees bound, that is,

(8) $$\lim_{n\to \infty }n\frac{{\left\{E\left(\frac{\varphi^{\prime }{\left(\theta \right)}^{2}}{I(\theta )}\right)\right\}}^{2}}{nE\left(\frac{\varphi^{\prime }{\left(\theta \right)}^{2}}{I(\theta )}\right)+E\left[{\left\{\frac{{\left(\varphi^{\prime }\left(\theta \right)\lambda \left(\theta \right)/I\left(\theta \right)\right)}^{\prime }}{\lambda (\theta )}\right\}}^{2}\right]}\geq \lim_{n\to \infty }n\frac{{\left\{E(\varphi^{\prime }\left(\theta \right))\right\}}^{2}}{nE\left(I\left(\theta \right)\right)+E\left\{{\left(\frac{\lambda^{\prime }\left(\theta \right)}{\lambda (\theta )}\right)}^{2}\right\}}.$$

Denote Borovkov–Sakhanenko Bound (3), Van Trees Bound (5), Bobrovsky–Mayor–Wolf–Zakai Bound (4), and Bound (1) as ${\mathrm{BS}}_{n}$, ${\mathrm{VT}}_{n}$, ${\mathrm{BMZ}}_{n,h}$ and $N_{n,h}$, when sample size is *n* and perturbation is *h*, respectively. Then, (8) means

(9) $$\lim_{n\to \infty }\frac{{\mathrm{BS}}_{n}}{{\mathrm{VT}}_{n}}=\lim_{n\to \infty }\frac{n\times {\mathrm{BS}}_{n}}{n\times {\mathrm{VT}}_{n}}=\frac{\lim_{n\to \infty }n\times {\mathrm{BS}}_{n}}{\lim_{n\to \infty }n\times {\mathrm{VT}}_{n}}\geq 1.$$

Hence, from (9),

(10) $${\mathrm{BS}}_{n}\geq {\mathrm{VT}}_{n}$$

holds for a sufficiently large *n*. Moreover, for this large n∈N,

(11) $$\lim_{h\to 0}N_{n,h}={\mathrm{BS}}_{n}\geq \lim_{h\to 0}{\mathrm{BMZ}}_{n,h}={\mathrm{VT}}_{n}$$

under (B1) and (B2). Hence, if Inequality (8) is strict, then $N_{n,h}>{\mathrm{BMZ}}_{n,h}$ for this large n∈N and a sufficiently small *h* by (10) and (11). The equality in (8) holds if and only if $\varphi^{\prime }$ is proportional to I(θ). Therefore, Bound (1) is asymptotically superior to the Bobrovsky–Mayor–Wolf–Zakai bound (4) for a sufficiently small *h*.

However, the comparison between Bounds (1) and (4) is not easy for a finite *n*. Hence, we now show comparisons of various existing bounds in two simple examples for fixed n∈N and $h\in R^{1}$.

**Example** **1.** *Let $X_{1},\ldots ,X_{n}$ be a sequence of iid random variables according to N(θ,1)$\left(\theta \in \Theta =R^{1}\right)$. We show that Bound* (1) *is asymptotically tighter than Bobrovsky–Mayor–Wolf–Zakai Bound *(4)* for a sufficiently large n. Suppose that the prior of θ is $N(m,\tau^{2})$, where m and τ>0 are known constants. Denote $X=(X_{1},\ldots ,X_{n})$ and $x=(x_{1},\ldots ,x_{n})$. In this model, Fisher information I(θ) per observation equals 1. We consider the estimation problem for $\varphi \left(\theta \right)=\theta^{2}$ since Bound* (1) *coincides with Bound* (4) *for φ(θ)=θ (see also [5,6]).*

*First, we calculated Bobrovsky–Mayor–Wolf–Zakai Bound* (4). *The ratio of f(x,θ+h) and f(x,θ) is*

(12) $$\frac{f(x,\theta +h)}{f(x,\theta )}=\exp\left\{hT-\frac{n}{2}\left(2h\theta +h^{2}\right)-\frac{h}{2\tau^{2}}\left(2\theta -2m+h\right)\right\},$$

*where $T=\sum_{i=1}^{n}X_{i}$. Since the conditional distribution of T given θ is N(nθ,n) and the moment generating function $g_{T}\left(s\right)$ is*

(13) $$g_{T}\left(s\right)=\exp\left(sn\theta +\frac{s^{2}n}{2}\right),$$

*the conditional expectation $E_{T|\theta }\left\{\exp\left(2hT\right)\right\}$ is*

(14) $$E_{T|\theta }\left\{\exp\left(2hT\right)\right\}=g_{T}\left(2h\right)=\exp\left(2hn\theta +2h^{2}n\right),$$

*where $E_{T|\theta }\left(\cdot \right)$ denotes the conditional expectation with respect to the conditional distribution of T given θ. Then, from* (12) *and* (14), *we have that*

(15) $$\begin{array}{cc} E\left[{\left\{\frac{f(X,\theta +h)}{f(X,\theta )}\right\}}^{2}\right]= & E\left[\exp\left\{2hT-n\left(2h\theta +h^{2}\right)-\frac{h}{\tau^{2}}\left(2\theta -2m+h\right)\right\}\right]\\ = & E\left[E_{T|\theta }\left\{\exp(2hT)\right\}\exp\left\{-nh^{2}-\frac{h}{\tau^{2}}\left(-2m+h\right)-2h\theta \left(n+\frac{1}{\tau^{2}}\right)\right\}\right]\\ = & E\left[\exp\left\{nh^{2}+\frac{2hm}{\tau^{2}}-\frac{h^{2}}{\tau^{2}}\right\}\exp\left\{-\frac{2h\theta }{\tau^{2}}\right\}\right]\\ = & \exp\left\{nh^{2}+\frac{2hm}{\tau^{2}}-\frac{h^{2}}{\tau^{2}}\right\}\exp\left\{-\frac{2hm}{\tau^{2}}+\frac{2h^{2}}{\tau^{2}}\right\}\\ = & \exp\left(nh^{2}+\frac{h^{2}}{\tau^{2}}\right). \end{array}$$

*We can easily obtain E{φ(θ)−φ(θ−h)}=h(2θ−h). Hence, Bobrovsky–Mayer–Wolf–Zakai Bound* (4) *is equal to*

(16) $$\frac{{\left\{h\left(2m-h\right)\right\}}^{2}}{\exp\left\{h^{2}\left(n+\frac{1}{\tau^{2}}\right)\right\}-1}\;\left(={\mathrm{BMZ}}_{h},\mathrm{say}\right)$$

*from* (15). *Next, we calculated Bound* (1). *Since I(θ)=1, $\varphi \left(\theta \right)=\theta^{2}$ and $\varphi^{\prime }\left(\theta \right)=2\theta$,*

(17) $$E\left[\frac{\varphi^{\prime }\left(\theta \right)}{I(\theta )}\left\{\varphi \left(\theta \right)-\varphi \left(\theta -h\right)\right\}\right]=E\left(4\theta^{2}h-2\theta h^{2}\right)=2h\left\{2\left(m^{2}+\tau^{2}\right)-mh\right\}.$$

*Since*

(18) $$\frac{f(X,\theta +h)}{f(X,\theta )}\frac{\varphi^{\prime }\left(\theta +h\right)}{I(\theta +h)}=2\left(\theta +h\right)\exp\left\{hT-\frac{n}{2}\left(2h\theta +h^{2}\right)-\frac{h}{2\tau^{2}}\left(2\theta -2m+h\right)\right\},$$

*we have*

(19) $$\begin{array}{cc} \; & E\left[{\left\{\frac{f(X,\theta +h)}{f(X,\theta )}\frac{\varphi^{\prime }\left(\theta +h\right)}{I(\theta +h)}\right\}}^{2}\right]\\ = & E\left[4{\left(\theta +h\right)}^{2}E_{T|\theta }\left\{\exp\left(2hT\right)\right\}\exp\left\{-n\left(2h\theta +h^{2}\right)-\frac{h}{\tau^{2}}\left(2\theta -2m+h\right)\right\}\right]\\ = & 4\exp\left\{nh^{2}-\frac{h}{\tau^{2}}\left(-2m+h\right)\right\}E\left[{\left(\theta +h\right)}^{2}\exp\left(-\frac{2h}{\tau^{2}}\theta \right)\right]\; \end{array}$$

*from* (18) *and* (14). *Here, since moment-generating function $g_{\theta }\left(s\right)$ of θ is $g_{\theta }\left(s\right)=E\left\{\exp\left(s\theta \right)\right\}=\exp\left(sm+\frac{s^{2}\tau^{2}}{2}\right)$,*

(20) $$\begin{array}{cc} g_{\theta }^{\prime }\left(s\right)= & E\left\{\theta \exp(s\theta )\right\}=\left(m+s\tau^{2}\right)\exp\left(sm+\frac{s^{2}\tau^{2}}{2}\right),\\ g_{\theta }^{\prime \prime }\left(s\right)= & E\left\{\theta^{2}\exp\left(s\theta \right)\right\}=\left\{\tau^{2}+{\left(m+s\tau^{2}\right)}^{2}\right\}\exp\left(sm+\frac{s^{2}\tau^{2}}{2}\right). \end{array}$$

*So, from* (20), *we obtain*

(21) $$\begin{array}{cc} E\left\{\exp\left(-\frac{2h}{\tau^{2}}\theta \right)\right\}= & \exp\left\{-\frac{2hm}{\tau^{2}}+\frac{2h^{2}}{\tau^{2}}\right\},\\ E\left\{\theta \exp\left(-\frac{2h}{\tau^{2}}\theta \right)\right\}= & \left(m-2h\right)\exp\left\{-\frac{2hm}{\tau^{2}}+\frac{2h^{2}}{\tau^{2}}\right\},\\ E\left\{\theta^{2}\exp\left(-\frac{2h}{\tau^{2}}\theta \right)\right\}= & \left\{\tau^{2}+{\left(m-2h\right)}^{2}\right\}\exp\left\{-\frac{2hm}{\tau^{2}}+\frac{2h^{2}}{\tau^{2}}\right\}. \end{array}$$

*Hence, from* (19) *and* (21),

(22) $$E\left[{\left\{\frac{f(X,\theta +h)}{f(X,\theta )}\frac{\varphi^{\prime }\left(\theta +h\right)}{I(\theta +h)}\right\}}^{2}\right]=4\left\{\tau^{2}+{\left(m-h\right)}^{2}\right\}\exp\left(nh^{2}+\frac{h^{2}}{\tau^{2}}\right).$$

*Moreover, we have*

(23) $$\begin{array}{cc} E\left[\frac{f(X,\theta +h)}{f(X,\theta )}\frac{\varphi^{\prime }\left(\theta +h\right)}{I(\theta +h)}\frac{\varphi^{\prime }\left(\theta \right)}{I(\theta )}\right]= & 4E\left\{\theta \left(\theta +h\right)\frac{f(X,\theta +h)}{f(X,\theta )}\right\}\\ = & 4\;\int \int \;\theta \left(\theta +h\right)\frac{f(x,\theta +h)}{f(x,\theta )}f\left(x,\theta \right)d\theta d\mu \left(x\right)\\ = & 4\;\int \int \;(t-h)tf(x,t)dtd\mu (x)\;(\mathrm{substitute}\;t=\theta +h)\\ = & 4E\left\{\left(\theta -h\right)\theta \right\}=4\left(m^{2}+\tau^{2}-hm\right), \end{array}$$

*and*

(24) $$E\left[{\left\{\frac{\varphi^{\prime }\left(\theta \right)}{I(\theta )}\right\}}^{2}\right]=4E\left(\theta^{2}\right)=4\left(\tau^{2}+m^{2}\right).$$

*From* (22)–(24),

(25) $$E\left[{\left\{\frac{f(X,\theta +h)}{f(X,\theta )}\frac{\varphi^{\prime }\left(\theta +h\right)}{I(\theta +h)}-\frac{\varphi^{\prime }\left(\theta \right)}{I(\theta )}\right\}}^{2}\right]=4\left\{\tau^{2}+{\left(m-h\right)}^{2}\right\}\exp\left\{h^{2}\left(n+\frac{1}{\tau^{2}}\right)\right\}-4\left(m^{2}+\tau^{2}-2hm\right).$$

*Therefore, Bound* (1) *is equal to*

(26) $$\frac{{\left[h\left\{2(m^{2}+\tau^{2})-mh\right\}\right]}^{2}}{\left\{\tau^{2}+{\left(m-h\right)}^{2}\right\}\exp\left\{h^{2}\left(n+\frac{1}{\tau^{2}}\right)\right\}-\left(m^{2}+\tau^{2}-2hm\right)}\;\left(=N_{h},\;\mathrm{say}\right).$$

*Lastly, we compare* (1) *and* (4). *From Bounds* (1) *and* (4), *we have*

(27) $$E\left\{{\left(\hat{\varphi }\left(X\right)-\varphi \left(\theta \right)\right)}^{2}\right\}\geq {\mathrm{BMZ}}_{h},\;N_{h}$$

*for arbitrary $h\in R^{1}$. In general, while the Bayes risk is $O(n^{-1})$, bounds ${\mathrm{BMZ}}_{h}$ and $N_{h}$ are $O(\exp\left(-nh^{2}\right))$ or decrease exponentially for h≠0 as n→∞. Thus, we take the limit as h→0 in order to obtain an asymptotically tighter bound. Define $\lim_{h\to 0}{\mathrm{BMZ}}_{h}={\mathrm{BMZ}}_{0}$ and $\lim_{h\to 0}N_{h}=N_{0}$. Since*

(28) $${\mathrm{BMZ}}_{0}=\frac{4m^{2}}{n+\frac{1}{\tau^{2}}},\;\;N_{0}=\frac{4(m^{2}+\tau^{2})}{\frac{1}{m^{2}+\tau^{2}}+n+\frac{1}{\tau^{2}}}$$

*from* (16) *and* (26), *we may compare their reciprocals, $4/{\mathrm{BMZ}}_{0}$ and $4/N_{0}$, in order to compare ${\mathrm{BMZ}}_{0}$ and $N_{0}$. ${\mathrm{BMZ}}_{0}$ and $N_{0}$ are the Van Trees and Borovkov–Sakhanenko bounds, respectively. The Borovkov–Sakhanenko bound is asymptotically tighter than the Van Trees bound. In this case, the Borovkov–Sakhanenko bound is also tighter than the Van Trees bound for fixed n. In fact, since the difference is*

(29) $$\begin{array}{cc} \frac{4}{{\mathrm{BMZ}}_{0}}-\frac{4}{N_{0}} & =\frac{1}{m^{2}\left(m^{2}+\tau^{2}\right)}\left(\frac{m^{2}}{m^{2}+1}-n\tau^{2}-1\right)\\ \; & <\frac{1}{m^{2}\left(m^{2}+\tau^{2}\right)}\left(1-n\tau^{2}-1\right)=\frac{-n\tau^{2}}{m^{2}\left(m^{2}+\tau^{2}\right)}<0 \end{array}$$

*from* (28), *so $4/{\mathrm{BMZ}}_{0}>4/N_{0}$ and hence ${\mathrm{BMZ}}_{0}<N_{0}$ for all n∈N.*

*Next, we compare these bound to the Bayes risk of the Bayes estimator ${\hat{\varphi }}_{B}\left(X\right)$ of $\varphi \left(\theta \right)=\theta^{2}$. The Bayes estimator ${\hat{\varphi }}_{B}\left(X\right)$ is given by*

(30) $${\hat{\varphi }}_{B}\left(X\right)=\frac{1}{n+(1/\tau^{2})}+\frac{T+(m/\tau^{2})}{n+(1/\tau^{2})}.$$

*Then, the Bayes risk of* (30) *is*

(31) $$\begin{array}{cc} E\left\{{\left({\hat{\varphi }}_{B}\left(X\right)-\varphi \left(\theta \right)\right)}^{2}\right\}= & \frac{2\tau^{2}\left(2n\tau^{4}+2m^{2}\tau^{2}n+2m^{2}+\tau^{2}\right)}{{\left(n\tau^{2}+1\right)}^{2}}\\ = & 4\left(m^{2}+\tau^{2}\right)n^{-1}+\frac{-2(2m^{2}+3\tau^{2})}{\tau^{2}}n^{-2}+O\left(n^{-3}\right)\;\left(n\to \infty \right). \end{array}$$

*Then, the normalized risk satisfies*

(32) $$\lim_{n\to \infty }nE\left\{{\left({\hat{\varphi }}_{B}\left(X\right)-\varphi \left(\theta \right)\right)}^{2}\right\}=4\left(m^{2}+\tau^{2}\right)=\lim_{n\to \infty }nN_{0}>4m^{2}=\lim_{n\to \infty }n{\mathrm{BMZ}}_{0}.$$

*Thus, the Van Trees bound is not asymptotically tight, while the Borovkov–Sakhanenko bound is asymptotically tight.*

**Example** **2.** *We considered the Bernoulli logit model of Example 2 in [16] when the sample size was 1. Bound* (1) *was not always better than Bobrovsky–Mayor–Wolf–Zakai Bound* (4). *Let X have Bernoulli distribution $\mathrm{Ber}\left(\frac{e^{\theta }}{1+e^{\theta }}\right)$ ($\theta \in R^{1}$). Then, the probability density function of X given θ is*

(33) $$f\left(x\;|\;\theta \right)=e^{\theta x}\frac{1}{1+e^{\theta }}\;\left(x=0,1\right).$$

*It is assumed that the prior density of θ is the conjugate, a version of Type IV generalized logistic distribution (e.g., [18]); then,*

(34) $$\lambda \left(\theta \right)=30e^{3\theta }{\left(1+e^{\theta }\right)}^{-6}\;\left(\theta \in R^{1}\right).$$

*We set the hyperparameters to these values for some moment conditions. In this case, Fisher information for Model (33) is given by*

(35) $$I\left(\theta \right)=\frac{e^{\theta }}{{\left(1+e^{\theta }\right)}^{2}},$$

*and we considered the estimation problem of φ(θ)=θ.*

*In this example, we calculated Bound* (1) *in the first place. Combining* (33)–(35), *we have*

(36) $$\frac{f(x,\theta +h)}{f(x,\theta )}\frac{\varphi^{\prime }\left(\theta +h\right)}{I(\theta +h)}=e^{hx}\frac{{\left(1+e^{\theta }\right)}^{7}}{{\left(1+e^{\theta +h}\right)}^{5}}e^{2h-\theta }$$

*for $h\in R^{1}$. Since X given θ is distributed as $\mathrm{Ber}\left(\frac{e^{\theta }}{1+e^{\theta }}\right)$, it holds*

(37) $$E_{X|\theta }\left(e^{2hX}\right)=\frac{1+e^{\theta +2h}}{1+e^{\theta }},$$

*where $E_{X|\theta }\left(\cdot \right)$ means the expectation with respect to the conditional distribution of X given θ. Then, we have*

(38) $$\begin{array}{cc} E\left[{\left\{\frac{f(X,\theta +h)}{f(X,\theta )}\frac{\varphi^{\prime }\left(\theta +h\right)}{I(\theta +h)}\right\}}^{2}\right]= & E\left(E_{X|\theta }\left[{\left\{\frac{f(X,\theta +h)}{f(X,\theta )}\frac{1}{I(\theta +h)}\right\}}^{2}\right]\right)\\ = & E\left\{\frac{{\left(1+e^{\theta }\right)}^{14}}{{\left(1+e^{\theta +h}\right)}^{10}}e^{4h-2\theta }E_{X|\theta }\left(e^{2hX}\right)\right\}\\ = & E\left\{{\left(1+e^{\theta }\right)}^{13}{\left(1+e^{\theta +h}\right)}^{-10}\left(1+e^{\theta +2h}\right)e^{4h-2\theta }\right\}\\ = & 30e^{4h}\int_{-\infty }^{\infty }{\left(1+e^{\theta }\right)}^{7}\left(1+e^{\theta +2h}\right)e^{\theta }{\left(1+e^{\theta +h}\right)}^{-10}d\theta \\ = & \frac{5}{6}\left\{10\cosh(h)+10\cosh(2h)+10\cosh(3h)+\cosh(4h)+5\right\}, \end{array}$$

*by* (35) *and* (37), *where* $\cosh\left(x\right)=\frac{1}{2}\left(e^{x}+e^{-x}\right)$ *is the hyperbolic cosine. Moreover, we have*

(39) $$\begin{array}{cc} E\left[\frac{f(X,\theta +h)}{f(X,\theta )}\frac{\varphi^{\prime }\left(\theta +h\right)\varphi^{\prime }\left(\theta \right)}{I(\theta +h)I(\theta )}\right]= & \;\int \int \;f\left(x,\theta +h\right)\frac{1}{I(\theta +h)I(\theta )}d\theta dF\left(x\right)\\ = & \;\int \int \;f\left(x,t\right)\frac{1}{I(t)I(t-h)}dtdF\left(x\right)\;\left(\mathrm{substitute}\;t=\theta +h\right)\\ = & \int \lambda \left(t\right)\frac{1}{I(t)I(t-h)}dt\\ = & 30\int {\left(1+e^{t}\right)}^{-4}{\left(1+e^{t-h}\right)}^{2}e^{t+h}dt\\ = & 10\left\{1+2\cosh(h)\right\}, \end{array}$$

*where F(·) is the cumulative distribution function of $\mathrm{Ber}\left(\frac{e^{\theta }}{1+e^{\theta }}\right)$. In a similar way, we have*

(40) $$E\left\{\frac{\varphi^{\prime }{\left(\theta \right)}^{2}}{I{\left(\theta \right)}^{2}}\right\}=E\left\{\frac{{\left(1+e^{\theta }\right)}^{4}}{e^{2\theta }}\right\}=30\int {\left(1+e^{\theta }\right)}^{-2}e^{\theta }d\theta =30$$

*and*

(41) $$E\left[\left\{\varphi (\theta )-\varphi (\theta -h)\right\}\frac{\varphi^{\prime }\left(\theta \right)}{I(\theta )}\right]=E\left\{\frac{h}{I(\theta )}\right\}=30h\int e^{2\theta }{\left(1+e^{\theta }\right)}^{-4}d\theta =5h.$$

*Hence, we can show from* (38)–(41) *that the right-hand side of* (1) *equals*

(42) $$\begin{array}{cc} \; & \frac{{\left(E\left[\left\{\varphi (\theta )-\varphi (\theta -h)\right\}\frac{\varphi^{\prime }\left(\theta \right)}{I(\theta )}\right]\right)}^{2}}{E\left[{\left\{\frac{f(X,\theta +h)}{f(X,\theta )}\frac{\varphi^{\prime }\left(\theta +h\right)}{I(\theta +h)}\right\}}^{2}\right]-2E\left[\frac{f(X,\theta +h)}{f(X,\theta )}\frac{\varphi^{\prime }\left(\theta +h\right)\varphi^{\prime }\left(\theta \right)}{I(\theta +h)I(\theta )}\right]+E\left\{\frac{\varphi^{\prime }{\left(\theta \right)}^{2}}{I{\left(\theta \right)}^{2}}\right\}}\\ = & \frac{{\left(5h\right)}^{2}}{\frac{10}{3}\sinh{\left(\frac{h}{2}\right)}^{2}\left\{33\cosh(h)+12\cosh(2h)+\cosh(3h)+8\right\}}\\ = & \frac{30h^{2}}{-38\cosh(h)+10\cosh(2h)+10\cosh(3h)+\cosh(4h)+17}\;(=:\left(N_{h},\mathrm{say}\right), \end{array}$$

*where $\sinh\left(x\right)=\frac{1}{2}\left(e^{x}-e^{-x}\right)$ is the hyperbolic sine. The Borovkov–Sakhanenko bound (3) is calculated as*

(43) $$N_{0}=\lim_{h\to 0}N_{h}=\frac{5}{9}\approx 0.556.$$

*In the second place, we calculate Bound* (4). *In a similar way to* (38) *and* (39), *we have*

(44) $$\begin{array}{cc} E\left[{\left\{\frac{f(X,\theta +h)}{f(X,\theta )}\right\}}^{2}\right]= & E\left\{\left(1+e^{\theta +2h}\right){\left(1+e^{\theta }\right)}^{13}{\left(1+e^{\theta +h}\right)}^{-14}e^{6h}\right\}\\ = & 30e^{6h}\int \left(1+e^{\theta +2h}\right){\left(1+e^{\theta }\right)}^{7}{\left(1+e^{\theta +h}\right)}^{-14}e^{3\theta }d\theta \\ = & \frac{1}{858}\left\{318\cosh(h)+231\cosh(2h)+116\cosh(3h)+18\cosh(4h)+175\right\} \end{array}$$

*and E{φ(θ)−φ(θ−h)}=h. Hence, by substituting* (44) *into* (4), *we have*

(45) $$\begin{array}{cc} \; & \frac{{\left[E\{\varphi (\theta )-\varphi (\theta -h)\}\right]}^{2}}{E\left[{\left\{\frac{f(X,\theta +h)}{f(X,\theta )}\right\}}^{2}\right]-1}\\ = & \frac{h^{2}}{\frac{1}{858}\left\{318\cosh(h)+231\cosh(2h)+116\cosh(3h)+18\cosh(4h)+175\right\}-1}\\ = & \frac{858h^{2}}{318\cosh(h)+231\cosh(2h)+116\cosh(3h)+18\cosh(4h)-683}\;\left(=:{\mathrm{BMZ}}_{h},\mathrm{say}\right). \end{array}$$

*The Van Trees bound is calculated as*

(46) $${\mathrm{BMZ}}_{0}=\lim_{h\to 0}{\mathrm{BMZ}}_{h}=\frac{2}{3}\approx 0.667.$$

*In last place, we compute the Bayes risk of the Bayes estimator ${\hat{\theta }}_{B}\left(X\right)$ of θ, as follows. Since the posterior density of θ, given X=x, is given by*

(47) $$60e^{\theta (x+3)}{\left(1+e^{\theta }\right)}^{-7}\;\left(0<\theta <1\right),$$

*the Bayes estimator is calculated as ${\hat{\theta }}_{B}\left(0\right)=E\left(\theta |X=0\right)=60\int_{-\infty }^{\infty }\theta e^{3\theta }{\left(1+e^{\theta }\right)}^{-7}d\theta =-1/3$, ${\hat{\theta }}_{B}\left(1\right)=E\left(\theta |X=1\right)=60\int_{-\infty }^{\infty }\theta e^{4\theta }{\left(1+e^{\theta }\right)}^{-7}d\theta =1/3$. Then, by easy but tedious calculation, the Bayes risk of ${\hat{\theta }}_{B}$ is*

(48) $$E\left\{{\left({\hat{\theta }}_{B}-\theta \right)}^{2}\right\}=\frac{\pi^{2}}{3}-\frac{47}{18}\approx 0.679.$$

**Figure 1:**
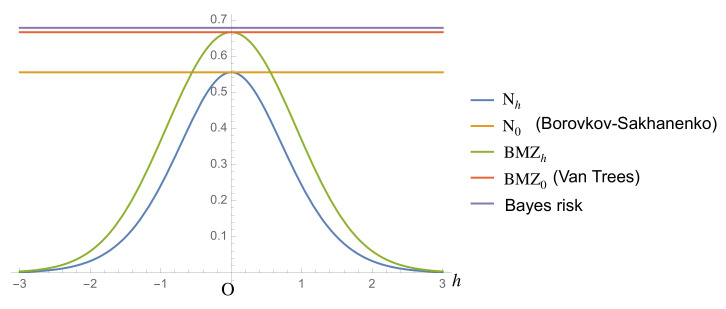
$N_{h}$, $N_{0}$, ${\mathrm{BMZ}}_{h}$, ${\mathrm{BMZ}}_{0}$, and Bayes risk.

*Then, we can plot the values of $N_{h}$, $N_{0}$, ${\mathrm{BMZ}}_{h}$, ${\mathrm{BMZ}}_{0}$ and the Bayes risk of ${\hat{\theta }}_{B}$ from* (42)–(46) *and* (48) *(Figure 1). Figure 1 shows that Bound* (1) *is lower than Bound* (4) *for any h under Prior* (34) *when the sample size equals 1. However, in Section 3, we show by using the Laplace method that Bound* (1) *is tighter than Bound* (4) *for a large sample size.*

## 3. Asymptotic Comparison by Laplace Approximation

In this section, we consider Example 2 in the previous section, again in the case when sample size is *n*. Bound (1) is asymptotically better than Bound (4) for a sufficiently large *n* by using the Laplace method. These bounds are only approximations as n→∞. The probability density function of $X_{i}$ given θ is

(49) $$f\left(x_{i}\;|\;\theta \right)={\left(\frac{e^{\theta }}{1+e^{\theta }}\right)}^{x_{i}}{\left(\frac{1}{1+e^{\theta }}\right)}^{1-x_{i}}=e^{\theta x_{i}}\frac{1}{1+e^{\theta }}\;\left(x_{i}=0,1;\;\theta \in R^{1};\;i=1,\ldots ,n\right)$$

and the likelihood ratio of (49) is

(50) $$\frac{f(x_{i}\;|\;\theta +h)}{f(x_{i}\;|\;\theta )}=e^{\left(\theta +h\right)x_{i}}\frac{1}{1+e^{\theta +h}}e^{-\theta x_{i}}\left(1+e^{\theta }\right)=e^{hx_{i}}\frac{1+e^{\theta }}{1+e^{\theta +h}}\;\left(h\in R^{1}\right).$$

Assume that the prior density of θ is

(51) $$\lambda \left(\theta \right)=\frac{1}{B(c_{1},c_{2}-c_{1})}e^{c_{1}\theta }{\left(1+e^{\theta }\right)}^{-c_{2}}\;\left(\theta \in R^{1};\;c_{2}>c_{1}+1>2\right).$$

Then, the ratio of (51) is equal to

(52) $$\frac{\lambda (\theta +h)}{\lambda (\theta )}=e^{\left(\theta +h\right)c_{1}}{\left(1+e^{\theta +h}\right)}^{-c_{2}}e^{-\theta c_{1}}{\left(1+e^{\theta }\right)}^{c_{2}}=e^{c_{1}h}{\left(1+e^{\theta +h}\right)}^{-c_{2}}{\left(1+e^{\theta }\right)}^{c_{2}}.$$

By denoting $X=(X_{1},\ldots ,X_{n})$, and $x=(x_{1},\ldots ,x_{n})$, the ratio of joint probability density functions of (X,θ) is

(53) $$\begin{array}{cc} P:=\frac{f(x,\theta +h)}{f(x,\theta )}= & \left\{\prod_{i=1}^{n}\frac{f(x_{i}\;|\;\theta +h)}{f(x_{i}\;|\;\theta )}\right\}\frac{\lambda (\theta +h)}{\lambda (\theta )}=e^{h\sum_{i=1}^{n}x_{i}}{\left(1+e^{\theta }\right)}^{n+c_{2}}{\left(1+e^{\theta +h}\right)}^{-n-c_{2}}e^{c_{1}h} \end{array}$$

by the iid assumption of $X_{i}\;|\;\theta$, (50), and (52). From (53), we have

(54) $$\begin{array}{cc} E(P^{2})= & E\left[e^{2h\sum_{i=1}^{n}X_{i}}{\left(1+e^{\theta }\right)}^{2n+2c_{2}}{\left(1+e^{\theta +h}\right)}^{-2n-2c_{2}}e^{2c_{1}h}\right]\\ = & E\left[E_{X|\theta }\left(e^{2h\sum_{i=1}^{n}X_{i}}\right){\left(1+e^{\theta }\right)}^{2n+2c_{2}}{\left(1+e^{\theta +h}\right)}^{-2n-2c_{2}}e^{2c_{1}h}\right]\\ = & E\left[{\left\{E_{X|\theta }\left(e^{2hX_{1}}\right)\right\}}^{n}{\left(1+e^{\theta }\right)}^{2n+2c_{2}}{\left(1+e^{\theta +h}\right)}^{-2n-2c_{2}}e^{2c_{1}h}\right]. \end{array}$$

By (37), we have

(55) $$\begin{array}{cc} E(P^{2})= & E\left[{\left(1+e^{\theta }\right)}^{n+2c_{2}}{\left(1+e^{\theta +h}\right)}^{-2n-2c_{2}}{\left(1+e^{\theta +2h}\right)}^{n}e^{2c_{1}h}\right]\\ = & \frac{e^{2c_{1}h}}{B(c_{1},c_{2}-c_{1})}\times \int_{-\infty }^{\infty }{\left(1+e^{\theta }\right)}^{c_{2}}{\left(1+e^{\theta +h}\right)}^{-2c_{2}}e^{c_{1}\theta }{\left\{\left(1+e^{\theta }\right){\left(1+e^{\theta +h}\right)}^{-2}\left(1+e^{\theta +2h}\right)\right\}}^{n}d\theta . \end{array}$$

Here, we consider the Laplace approximation of integral

(56) $$I_{1}=\int_{-\infty }^{\infty }{\left(1+e^{\theta }\right)}^{c_{2}}{\left(1+e^{\theta +h}\right)}^{-2c_{2}}e^{c_{1}\theta }{\left\{\left(1+e^{\theta }\right){\left(1+e^{\theta +h}\right)}^{-2}\left(1+e^{\theta +2h}\right)\right\}}^{n}d\theta$$

(see e.g., [19]). $I_{1}$ can be expressed as

(57) $$I_{1}=\int_{-\infty }^{\infty }g_{1}\left(\theta \right)\exp\left\{nk(\theta )\right\}d\theta ,$$

where $g_{1}\left(\theta \right)={\left(1+e^{\theta }\right)}^{c_{2}}{\left(1+e^{\theta +h}\right)}^{-2c_{2}}e^{c_{1}\theta }$ and $k\left(\theta \right)=\log\left\{\left(1+e^{\theta }\right){\left(1+e^{\theta +h}\right)}^{-2}\left(1+e^{\theta +2h}\right)\right\}$.

Since

(58) $$k^{\prime }\left(\theta \right)=-\frac{e^{\theta }{\left(-1+e^{h}\right)}^{2}\left(-1+e^{\theta +h}\right)}{\left(1+e^{\theta }\right)\left(1+e^{\theta +h}\right)\left(1+e^{\theta +2h}\right)},$$

if $k^{\prime }\left(\theta \right)=0$, then θ=−h. *k* takes its maximum at θ=−h,

(59) $$k^{\prime \prime }\left(-h\right)=-\frac{\tanh{\left(\frac{h}{2}\right)}^{2}}{2}<0$$

and $k^{\prime \prime }\left(-h\right)\to 0$ (h→0). Therefore, the Laplace approximation of $I_{1}$ gives

(60) $$I_{1}∼\exp\left\{nk(-h)\right\}g_{1}\left(-h\right)\sqrt{\frac{2\pi }{-nk^{\prime \prime }\left(-h\right)}}$$

as n→∞ from (57)–(59). Here, we have $k\left(-h\right)=\log\left\{\left(1+e^{-h}\right)\left(1+e^{h}\right)/4\right\}\geq 0$ since $e^{-h}+e^{h}\geq 2$ from the arithmetic-geometric mean inequality. The equality holds if and only if h=0. Hence, the leading term of Bobrovsky–Mayor–Wolf–Zakai Bound (4) is

(61) $$\frac{h^{2}}{\frac{e^{2c_{1}h}}{B(c_{1},c_{2}-c_{1})}J_{n}\left(-h\right)}$$

as n→∞, from (55) and (60), where

(62) $$J_{n}\left(-h\right)=\exp\left\{nk(-h)\right\}g_{1}\left(-h\right)\sqrt{\frac{2\pi }{-nk^{\prime \prime }\left(-h\right)}}.$$

In a similar way to the above, defining

(63) $$Q:=\frac{f(x,\theta +h)}{f(x,\theta )}\frac{\varphi^{\prime }\left(\theta +h\right)}{I(\theta +h)}=P\frac{{\left(1+e^{\theta +h}\right)}^{2}}{e^{\theta +h}},$$

we calculate

(64) $$\begin{array}{cc} E(Q^{2})= & E\left[{\left(1+e^{\theta }\right)}^{n+2c_{2}}{\left(1+e^{\theta +h}\right)}^{-2n-2c_{2}+4}{\left(1+e^{\theta +2h}\right)}^{n}e^{2c_{1}h-2\theta -2h}\right]\\ = & \frac{e^{2(c_{1}-1)h}}{B(c_{1},c_{2}-c_{1})}\times \int_{-\infty }^{\infty }{\left(1+e^{\theta }\right)}^{c_{2}}{\left(1+e^{\theta +h}\right)}^{-2c_{2}+4}e^{(c_{1}-2)\theta }{\left\{\left(1+e^{\theta }\right){\left(1+e^{\theta +h}\right)}^{-2}\left(1+e^{\theta +2h}\right)\right\}}^{n}d\theta . \end{array}$$

Here, we consider the Laplace approximation of the integral

(65) $$I_{2}=\int_{-\infty }^{\infty }g_{2}\left(\theta \right)\exp\left\{nk\left(\theta \right)\right\}d\theta ,$$

where $g_{2}\left(\theta \right)={\left(1+e^{\theta }\right)}^{c_{2}}{\left(1+e^{\theta +h}\right)}^{-2c_{2}+4}e^{(c_{1}-2)\theta }$ and k(θ) is defined in (57). The Laplace approximation of $I_{2}$ gives

(66) $$I_{2}∼\exp\left\{nk(-h)\right\}g_{2}\left(-h\right)\sqrt{\frac{2\pi }{-nk^{\prime \prime }\left(-h\right)}}=2^{4}e^{2h}J_{n}\left(-h\right)$$

as n→∞. Similarly to (41), we have

(67) $$\begin{array}{cc} E\left[\left\{\varphi (\theta )-\varphi (\theta -h)\right\}\frac{\varphi^{\prime }\left(\theta \right)}{I(\theta )}\right]= & E\left\{h\frac{{\left(1+e^{\theta }\right)}^{2}}{e^{\theta }}\right\}\\ = & \frac{h}{B(c_{1},c_{2}-c_{1})}\int_{-\infty }^{\infty }e^{(c_{1}-1)\theta }{\left(1+e^{\theta }\right)}^{-c_{2}+2}d\theta \\ = & \frac{h}{B(c_{1},c_{2}-c_{1})}\int_{0}^{1}t^{c_{1}-2}{\left(1-t\right)}^{c_{2}-c_{1}-2}dt\;\;\;\left(\mathrm{substitute}\;\;t=e^{\theta }/\left(1+e^{\theta }\right)\right)\\ = & h\frac{B(c_{1}-1,c_{2}-c_{1}-1)}{B(c_{1},c_{2}-c_{1})}. \end{array}$$

Hence, by using (64)–(67), the leading term of Bound (1) is

(68) $$\frac{{\left\{h\frac{B(c_{1}-1,c_{2}-c_{1}-1)}{B(c_{1},c_{2}-c_{1})}\right\}}^{2}}{\frac{2^{4}e^{2c_{1}h}}{B(c_{1},c_{2}-c_{1})}J_{n}\left(-h\right)}$$

as n→∞. Dividing (61) by (68) yields

(69) $$\begin{array}{cc} \frac{h^{2}/\left\{\frac{e^{2c_{1}h}}{B(c_{1},c_{2}-c_{1})}J_{n}\left(-h\right)\right\}}{{\left\{h\frac{B(c_{1}-1,c_{2}-c_{1}-1)}{B(c_{1},c_{2}-c_{1})}\right\}}^{2}/\left\{\frac{2^{4}e^{2c_{1}h}}{B(c_{1},c_{2}-c_{1})}J_{n}\left(-h\right)\right\}}= & {\left\{4\frac{B(c_{1},c_{2}-c_{1})}{B(c_{1}-1,c_{2}-c_{1}-1)}\right\}}^{2}\\ = & {\left\{4\frac{\left(c_{1}-1\right)\left(c_{2}-c_{1}-1\right)}{\left(c_{2}-2\right)\left(c_{2}-1\right)}\right\}}^{2}\\ < & {\left(\frac{c_{2}-2}{c_{2}-1}\right)}^{2}<1. \end{array}$$

The second inequality from the end follows from $\left(c_{2}-2\right)/2=\left\{\left(c_{1}-1\right)+\left(c_{2}-c_{1}-1\right)\right\}/2\geq \sqrt{\left(c_{1}-1\right)\left(c_{2}-c_{1}-1\right)}$ by the arithmetic-geometric mean inequality. Hence, (68) is asymptotically greater than (61) for any *h* in this setting.

## 4. Conclusions

Bayesian Cramér–Rao-type bounds are often useful for issues of asymptotical efficiency of estimators (for example, [4]). However the Borovkov–Sakhanenko bound is asymptotically tighter ([14,15]) than the Van Trees bound [1]. Since the Bobrovsky–Mayer–Wolf–Zakai bound [17] and the new bound in this paper converge to the Van Trees and Borovkov–Sakhanenko bounds, respectively, as h→0 under some conditions, it is natural to consider that their asymptotical property still holds for a small *h*. Examples in this paper supported this result. The new bound gives an asymptotic lower bound of normalized Bayes risk, and the bound cannot be improved as h→0.

## Appendix A. Regularity Conditions

We need the following conditions for the convergence of Bounds (1) and (2) to Borovkov–Sakhanenko and the Van Trees bounds, respectively.

(B1) There exist $h_{1}>0$ and a function $b_{1}\left(x,\theta \right)$, such that
  $$\begin{array}{c} E\left\{b_{1}^{2}\left(X,\theta \right)\right\}<\infty \;\;\mathrm{and}\;\;\left|\frac{\frac{f(x,\theta +h)}{f(x,\theta )}\frac{\varphi^{\prime }\left(\theta +h\right)}{I(\theta +h)}-\frac{\varphi^{\prime }\left(\theta \right)}{I(\theta )}}{h}\right|\leq b_{1}\left(x,\theta \right)\;\;\mathrm{for}\;\mathrm{all}\;\left|h\right|\leq h_{1}\;\;\mathrm{and}\;\mathrm{arbitrary}\;\theta \in \Theta . \end{array}$$
(B2) There exist $h_{2}>0$ and a function $b_{2}\left(x,\theta \right)$ such that
  $$E\left\{b_{2}^{2}\left(X,\theta \right)\right\}<\infty \;\;\mathrm{and}\;\;\left|\frac{\frac{f(x,\theta +h)}{f(x,\theta )}-1}{h}\right|\leq b_{2}\left(x,\theta \right)\;\;\mathrm{for}\;\mathrm{all}\;\left|h\right|\leq h_{2}\;\;\mathrm{and}\;\mathrm{arbitrary}\;\theta \in \Theta .$$

## Appendix B. Proof of Theorem 1

Theorem 1 is directly derived from [7]. However, we prove it here for the sake of clarity.

Let $G_{h}=\frac{1}{h}\left(\frac{f(x,\theta +h)}{f(x,\theta )}\frac{\varphi^{\prime }\left(\theta +h\right)}{I(\theta +h)}-\frac{\varphi^{\prime }\left(\theta \right)}{I(\theta )}\right)$. Then, we have

$$\begin{array}{cc} E(G_{h})= & \frac{1}{h}\left\{\;\int \int \;f\left(x,\theta +h\right)\frac{\varphi^{\prime }\left(\theta +h\right)}{I(\theta +h)}d\theta d\mu \left(x\right)-\;\int \int \;f\left(x,\theta \right)\frac{\varphi^{\prime }\left(\theta \right)}{I(\theta )}d\theta d\mu \left(x\right)\right\}\\ = & \frac{1}{h}\left\{\;\int \int \;f\left(x,t\right)\frac{\varphi^{\prime }\left(t\right)}{I(t)}dtd\mu \left(x\right)-\;\int \int \;f\left(x,\theta \right)\frac{\varphi^{\prime }\left(\theta \right)}{I(\theta )}d\theta d\mu \left(x\right)\right\}\;\left(\mathrm{substitute}\;t=\theta -h\right)\\ = & 0, \end{array}$$

and

$$\begin{array}{cc} E\{G_{h}\varphi \left(\theta \right)\}= & \frac{1}{h}\left\{\;\int \int \;\varphi \left(\theta \right)f\left(x,\theta +h\right)\frac{\varphi^{\prime }\left(\theta +h\right)}{I(\theta +h)}d\theta d\mu \left(x\right)-\;\int \int \;\varphi \left(\theta \right)f\left(x,\theta \right)\frac{\varphi^{\prime }\left(\theta \right)}{I(\theta )}d\theta d\mu \left(x\right)\right\}\\ = & \frac{1}{h}\left\{\;\int \int \;\varphi \left(t-h\right)f\left(x,t\right)\frac{\varphi^{\prime }\left(t\right)}{I(t)}dtd\mu \left(x\right)-\;\int \int \;\varphi \left(\theta \right)f\left(x,\theta \right)\frac{\varphi^{\prime }\left(\theta \right)}{I(\theta )}d\theta d\mu \left(x\right)\right\}\\ \; & \;(\mathrm{substitute}\;t=\theta -h)\\ = & \frac{1}{h}\left[E\left\{\varphi \left(\theta -h\right)\frac{\varphi^{\prime }\left(\theta \right)}{I(\theta )}\right\}-E\left\{\varphi \left(\theta \right)\frac{\varphi^{\prime }\left(\theta \right)}{I(\theta )}\right\}\right]\\ = & \frac{1}{h}E\left[\left\{\varphi \left(\theta -h\right)-\varphi \left(\theta \right)\right\}\frac{\varphi^{\prime }\left(\theta \right)}{I(\theta )}\right]. \end{array}$$

By Fubini’s theorem,

$$\begin{array}{cc} E\{G_{h}\hat{\varphi }\left(X\right)\}= & \frac{1}{h}\left\{\int \hat{\varphi }\left(x\right)\left(\int f\left(x,\theta +h\right)\frac{\varphi^{\prime }\left(\theta +h\right)}{I(\theta +h)}d\theta \right)d\mu \left(x\right)-\int \hat{\varphi }\left(x\right)\left(\int f\left(x,\theta \right)\frac{\varphi^{\prime }\left(\theta \right)}{I(\theta )}d\theta \right)d\mu \left(x\right)\right\}\\ = & \frac{1}{h}\left\{\int \hat{\varphi }\left(x\right)\left(\int f\left(x,t\right)\frac{\varphi^{\prime }\left(t\right)}{I(t)}dt\right)d\mu \left(x\right)-\int \hat{\varphi }\left(x\right)\left(\int f\left(x,\theta \right)\frac{\varphi^{\prime }\left(\theta \right)}{I(\theta )}d\theta \right)d\mu \left(x\right)\right\}\\ \; & \;(\mathrm{substitute}\;t=\theta -h)\\ = & 0, \end{array}$$

hence, from (A1), (A2), and (A3),

$$\begin{array}{c} \mathrm{Cov}\left(G_{h},\hat{\varphi }\left(X\right)-\varphi \left(\theta \right)\right)=\frac{1}{h}E\left[\left\{\varphi \left(\theta \right)-\varphi \left(\theta -h\right)\right\}\frac{\varphi^{\prime }\left(\theta \right)}{I(\theta )}\right]. \end{array}$$

From variance–covariance inequality, (A4) gives

(A5) $$E\left\{{\left(\hat{\varphi }\left(X\right)-\varphi \left(\theta \right)\right)}^{2}\right\}\geq \frac{{\left\{\mathrm{Cov}(G_{h},\hat{\varphi }\left(X\right)-\varphi \left(\theta \right))\right\}}^{2}}{E(G_{h}^{2})}=\frac{{\left\{E\left[\left\{\varphi \left(\theta \right)-\varphi \left(\theta -h\right)\right\}\frac{\varphi^{\prime }\left(\theta \right)}{I(\theta )}\right]\right\}}^{2}}{E\left[{\left\{\frac{f(X,\theta +h)}{f(X,\theta )}\frac{\varphi^{\prime }\left(\theta +h\right)}{I(\theta +h)}-\frac{\varphi^{\prime }\left(\theta \right)}{I(\theta )}\right\}}^{2}\right]},\;$$

which is the desired inequality.

## References

[1] Van Trees H.L. (1968). Detection, Estimation, and Modulation Theory, Part I.

[2] Borovkov A.A., Sakhanenko A.U. (1980). On estimates of the expected quadratic risk (in Russian). Probab. Math. Statist..

[3] Borovkov A.A. (1998). Mathematical Statistics.

[4] Gill R.D., Levit B.Y. (1995). Applications of the van Trees inequality: A Bayesian Cramér-Rao bound. Bernoulli.

[5] Koike K. (2006). An integral Bhattacharyya type bound for the Bayes risk. Commun. Stat. Theory Methods.

[6] Hashimoto S., Koike K. (2015). Bhattacharyya type information inequality for the Bayes risk. Commun. Stat. Theory Methods.

[7] Weinstein E., Weiss A.J. (1988). A general class of lower bounds in parameter estimation. IEEE Trans. Inf. Theory.

[8] Renaux A., Forster P., Larzabal P., Richmond C.D., Nehorai A. (2008). A fresh look at the Bayesian bounds of the Weiss-Weinstein family. IEEE Trans. Signal Process..

[9] Todros K., Tabrikian J. (2010). General classes of performance lower bounds for parameter estimation—Part II: Bayesian bounds. IEEE Trans. Inf. Theory.

[10] Ziv J., Zakai M. (1969). Some lower bounds on signal parameter estimation. IEEE Trans. Inf. Theory.

[11] Bell K.L., Steinberg Y., Ephraim Y., Van Trees H.L. (1997). Extended Ziv-Zakai lower bound for vector parameter estimation. IEEE Trans. Inf. Theory.

[12] Routtenberg T., Tabrikian J. (2012). A general class of outage error probability lower bounds in Bayesian parameter estimation. IEEE Trans. Signal Process..

[13] Van Trees H.L., Bell K.L. (2007). Bayesian Bounds for Parameter Estimation and Nonlinear Filtering/Tracking.

[14] Abu-Shanab R., Veretennikov A.Y. (2015). On asymptotic Borovkov-Sakhanenko inequality with unbounded parameter set. Theory Probab. Math. Stat..

[15] Koike K. (2020). Asymptotic comparison of some Bayesian information bounds. Commun. Stat. Theory Methods.

[16] Koike K. (2019). Attainments of the Bayesian information bounds. Commun. Stat. Theory Methods.

[17] Bobrovsky B.Z., Mayer-Wolf E., Zakai M. (1987). Some classes of grobal Cramér-Rao bounds. Ann. Stat..

[18] Prentice R.L. (1976). A generalization of the probit and logit models for dose response curves. Biometrics.

[19] Small C.G. (2010). Expansions and Asymptotics for Statistics.

